# Supplementary material for: MorphoNet 2.0: An innovative approach for qualitative assessment and segmentation curation of large-scale 3D time-lapse imaging datasets
Source: eLife. 2025 Dec 2;14:RP106227. doi: 10.7554/eLife.106227 (PMC12671910; doi:10.7554/eLife.106227)
Supplement: Supplementary file 1. [file elife-106227-supp1.docx]

**
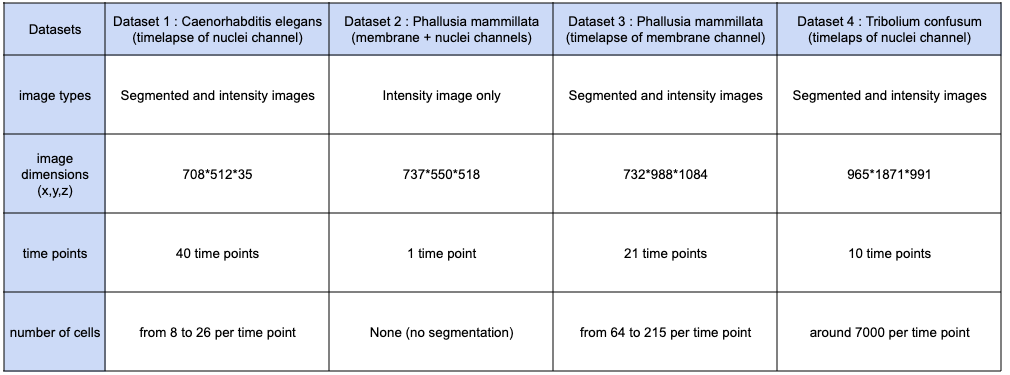
**

**
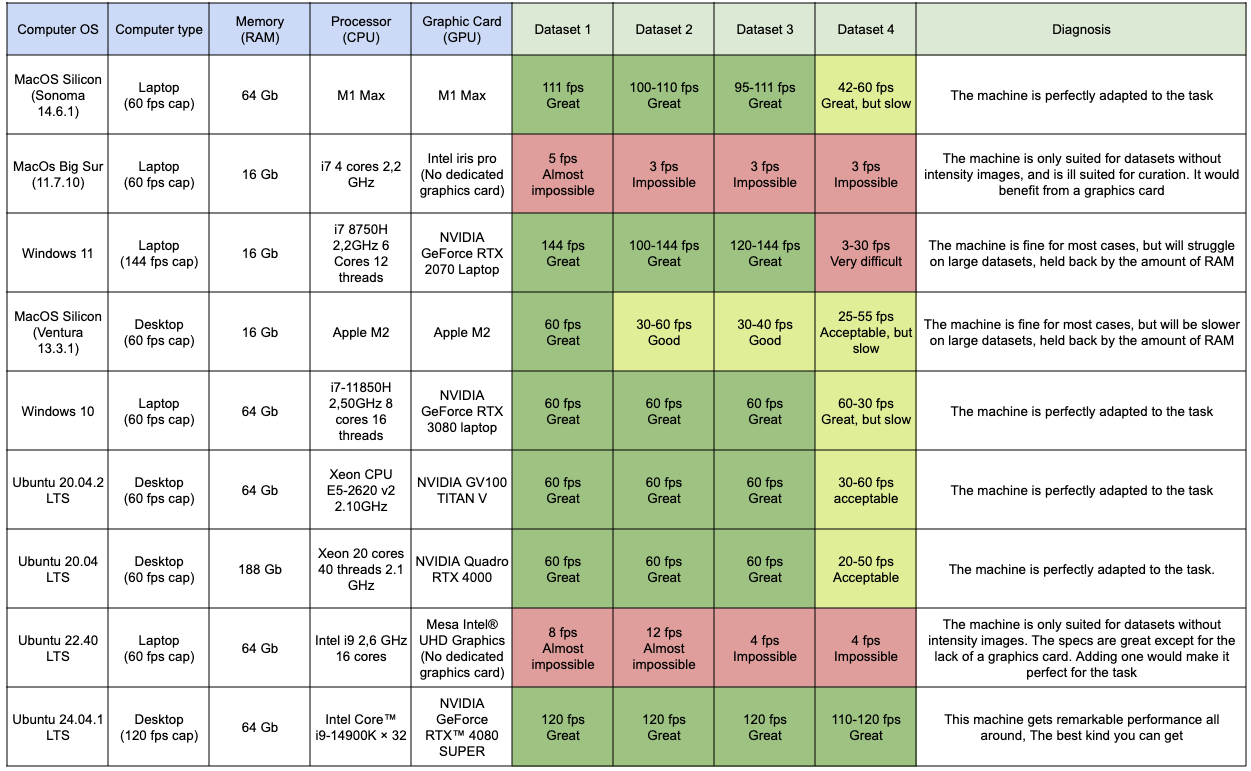
**

**Table 1**: *Performance of the MorphoNet standalone application of various devices. The upper table describes the datasets used for benchmarking the application. The lower table gives an evaluation of performances for different devices, as well as a description of the device’s specifications. The cells are colored according to whether or not a particular device is adapted for the visualization and/or curation of a particular dataset. The datasets can be accessed at the link below:* <https://doi.org/10.6084/m9.figshare.30529745.v1> in the BENCHMARKING_DATASETS folder.
