# Supplementary material for: MorphoNet 2.0: An innovative approach for qualitative assessment and segmentation curation of large-scale 3D time-lapse imaging datasets
Source: eLife. 2025 Dec 2;14:RP106227. doi: 10.7554/eLife.106227 (PMC12671910; doi:10.7554/eLife.106227)
Supplement: Supplementary file 2. [file elife-106227-supp2.docx]

| **I am:** | **I want to:** | **Related documentation:** |
| --- | --- | --- |
| A biologist | Visualize a dataset **located on the MorphoNet servers** (public or private) | 1. You can either :           - [Install the standalone app](https://morphonet.org/help_standalone) (handles large datasets)  OR    - [Use the website](https://morphonet.org/help_website) (handles small datasets)  2. [Use the MorphoNet 3D Viewer](https://morphonet.org/help_app?menu=app_general) |
|  | Visualize a dataset with **segmented** and/or **intensity** images on my computer | 1. [Create and view local datasets on MorphoNet standalone](https://morphonet.org/help_standalone#add_local)  2. [Use the MorphoNet 3D Viewer](https://morphonet.org/help_app?menu=app_general) |
|  | Curate a dataset with **segmented** and optionally **intensity** images on my computer | 1. [Create and view local datasets on MorphoNet standalone](https://morphonet.org/help_standalone#add_local)  2. [Look at a curation example](https://morphonet.org/help_curation?menu=curation)  3. [Have a look at all available default plugins](https://morphonet.org/help_app?menu=curations)  4. [List of automatically computed image properties](https://morphonet.org/help_curation?menu=regionproperties) 5. [Use the MorphoNet 3D Viewer](https://morphonet.org/help_app?menu=app_general) |
|  | Create and segment a dataset with **intensity images** only | 1. [Create a local datasets from your intensity images only on MorphoNet standalone](https://morphonet.org/help_standalone#add_local)  2. [Use the MorphoNet 3D Viewer](https://morphonet.org/help_app?menu=app_general) |
|  | Upload a dataset with **segmented** and/or **intensity** images on the MorphoNet server | You can either :            - [Upload a dataset from the MorphoNet application](https://morphonet.org/help_standalone#upload_dataset)  OR.    - [Upload a dataset with the FIJI plugin](https://morphonet.org/help_fiji?menu=fiji_basic#convert_upload) |
|  | Upload a dataset with **meshes** | 1. Look first at the [required mesh format](https://morphonet.org/help_format)  2. [Upload a dataset with meshes with the FIJI plugin](https://morphonet.org/help_fiji?menu=fiji_basic#upload_mesh) |
|  | **Share** a dataset on MorphoNet | [Share a dataset on the website](https://morphonet.org/help_website#share_dataset) |
|  | Add **properties** to your local dataset | 1. Have a look at the [Properties format](https://morphonet.org/help_format#property_types)  2. You can [use the MorphoNet 3D Viewer](https://morphonet.org/help_app?menu=info) to add a property from the menu |
|  | Add **properties** to the dataset located on the MorphoNet server | 1. Have a look at the [Properties format](https://morphonet.org/help_format#property_types)  2. [Upload properties with th FIJI plugin](https://morphonet.org/help_fiji?menu=fiji_basic#upload_properties) |
|  | Add and visualize **genetic** properties on a dataset | 1. [Create a genetic property](https://morphonet.org/help_format#genetic_property)  2. Add them using the [MorphoNet 3D Viewer](https://morphonet.org/help_app?menu=app_general) 3. and then use them in [Genetic menu](https://morphonet.org/help_app?menu=genetic) |
|  | **Download** a full dataset from a dataset | [Download the dataset from MorphoNet](https://morphonet.org/help_website#export_dataset) |
|  | Visualize and Interact with a dataset in **Virtual Reality** | 1. [Install the standalone app](https://morphonet.org/help_standalone) on Windows  2. Open the [Virtual Reality](https://morphonet.org/help_vr) mode |
|  |  |  |
| A **bio-image analyst** | Upload a dataset with **segmented** and/or **intensity** images on the MorphoNet server | Using the [API](https://morphonet.org/help_api) you can [convert your segmented images in meshes](https://morphonet.org/help_api?menu=morphonetnet#convert_in_meshes) and then directly [upload the meshes](https://morphonet.org/help_api?menu=morphonetnet#manage_meshes) . You can look at this [example](https://morphonet.org/helpfiles/API/Notebooks/UploadExample.html) from the [documentation](https://morphonet.org/helpfiles/API/Notebooks/MorphoNetNet.html#Create-your-dataset) |
|  | Upload a dataset with **meshes** | 1. Look first at the [required mesh format](https://morphonet.org/help_format)  2. [Upload the meshes](https://morphonet.org/help_api?menu=morphonetnet#manage_meshes) using the [API](https://morphonet.org/help_api) . You can look at this [example](https://morphonet.org/helpfiles/API/Notebooks/UploadExample.html) from the [documentation](https://morphonet.org/helpfiles/API/Notebooks/MorphoNetNet.html#Create-your-dataset) |
|  | **Share** a dataset on MorphoNet | [Share a dataset with the python API](https://morphonet.org/helpfiles/API/Notebooks/MorphoNetNet.html) |
|  | Add **properties** to the dataset located on the MorphoNet server | 1. Have a look at the [Properties format](https://morphonet.org/help_format#property_types)  2. [Upload properties with the python API](https://morphonet.org/helpfiles/API/Notebooks/MorphoNetNet.html#Manage-Properties) |
|  | **Download** a full dataset from a dataset | Download the meshes and the properties with the [python API](https://morphonet.org/helpfiles/API/morphonet.pagenet.html) |
|  | Create custom **plugins** for MorphoNet | 1. Look how to use [python API](https://morphonet.org/helpfiles/API/index.html) with the Standalone application in developer mode.  2. [Create custom plugins for bio-curation](https://morphonet.org/help_api?menu=morphonetplot#plugins) |
|  | Use MorphoNet to **analyze** 3D + t images | Look at the [MorphoNet tutorials in python](https://morphonet.org/help?menu=advanced) :         - [Visualize/Analyze complex 3D+time.](https://colab.research.google.com/drive/17j8oBZxag_u-_ihTeb7BkRDTVd-Yd4bn?usp=sharing)          - [How to segment and track 3D cells.](https://colab.research.google.com/drive/1yAKKTXL6ZezFOLS8U4M2Ew4JIYr6bJxq?usp=sharing)          - [How to create a simple simulation.](https://colab.research.google.com/drive/11hrZdKUa0e7CelMyJAGITVTtESZwX1af?usp=sharing)          - [How cell adjacency relationships impact cell state transitions.](https://colab.research.google.com/drive/1XuTx6LzYuV1Z122n7sxUAzxs9oSpTgQ9?usp=sharing) |
|  |  |  |
| A **developer** | Contribute to the development of **MorphoNet 3D viewer** | Clone the [MorphoNet Unity GitLab Project](https://gitlab.inria.fr/MorphoNet/morphonet_unity/) |
|  | Contribute to the development of **MorphoNet python API** | Clone the [MorphoNet Python Project](https://gitlab.inria.fr/MorphoNet/morphonet_api) |
|  | Integrate MorphoNet into your **website** | Look at the [API REST](https://morphonet.org/morphoapi) to configure your own urls |

**Table 2**: *MorphoNet Documentation hub on various help pages depending on user types and use cases.*
